# DNA methylation signatures as biomarkers of socioeconomic position

**DOI:** 10.1093/eep/dvac027

**Published:** 2022-12-14

**Authors:** Meghna Rajaprakash, Lorraine T Dean, Meredith Palmore, Sara B Johnson, Joan Kaufman, Daniele M Fallin, Christine Ladd-Acosta

**Affiliations:** Department of Neurology and Developmental Medicine, Kennedy Krieger Institute, Baltimore, MD 21205, USA; Department of Pediatrics, Johns Hopkins University School of Medicine, Baltimore, MD 21287, USA; Department of Psychiatry, Johns Hopkins University School of Medicine, Baltimore, MD 21205, USA; Department of Psychiatry, Johns Hopkins University School of Medicine, Baltimore, MD 21205, USA; Department of Pediatrics, Johns Hopkins University School of Medicine, Baltimore, MD 21287, USA; Department of Epidemiology, Johns Hopkins Bloomberg School of Public Health, Baltimore, MD 21205, USA; Department of Population, Family and Reproductive Health, Johns Hopkins Bloomberg School of Public Health, Baltimore, MD 21205, USA; Department of Mental Health, Johns Hopkins Bloomberg School of Public Health, Baltimore, MD, USA; Department of Epidemiology, Johns Hopkins Bloomberg School of Public Health, Baltimore, MD 21205, USA; Wendy Klag Center for Autism and Developmental Disabilities, Johns Hopkins Bloomberg School of Public Health, Baltimore, MD 21205, USA; Rollins School of Public Health, Emory University, Atlanta, GA 30322, USA; Department of Epidemiology, Johns Hopkins Bloomberg School of Public Health, Baltimore, MD 21205, USA; Wendy Klag Center for Autism and Developmental Disabilities, Johns Hopkins Bloomberg School of Public Health, Baltimore, MD 21205, USA

**Keywords:** DNA methylation, biomarker, socioeconomic position, social epigenomics, EWAS

## Abstract

This review article provides a framework for the use of deoxyribonucleic acid (DNA) methylation (DNAm) biomarkers to study the biological embedding of socioeconomic position (SEP) and summarizes the latest developments in the area. It presents the emerging literature showing associations between individual- and neighborhood-level SEP exposures and DNAm across the life course. In contrast to questionnaire-based methods of assessing SEP, we suggest that DNAm biomarkers may offer an accessible metric to study questions about SEP and health outcomes, acting as a personal dosimeter of exposure. However, further work remains in standardizing SEP measures across studies and evaluating consistency across domains, tissue types, and time periods. Meta-analyses of epigenetic associations with SEP are offered as one approach to confirm the replication of DNAm loci across studies. The development of DNAm biomarkers of SEP would provide a method for examining its impact on health outcomes in a more robust way, increasing the rigor of epidemiological studies.

The term epigenetics is used to describe the study of mitotically heritable cellular material above the level of the DNA sequence that helps to regulate organismal development, gene expression, gene imprinting, genome stability, and other biological processes. Because of these functions, the amount and patterns of epigenetic material differ across cell types and across developmental and life course windows and may change in response to the environment. The most studied types of epigenetic material include DNA methylation (DNAm), histone tail modifications, and chromatin structure. DNAm involves the covalent addition of a methyl or hydroxymethyl group to cytosine nucleotides in DNA at CpG dinucleotides. DNAm is commonly used in epidemiologic studies because it is practical to obtain biosamples, it is stable when collected and stored at various conditions due to the covalent bond, and we understand the biological and inheritance properties well [1]. Because DNAm is integral to regulating cellular and organismal biological processes, many studies have focused on evaluating its role in disease mechanisms, often as a mediator of genetic or environmental risk factors. More recently, evidence has emerged showing that epigenetic changes, including DNAm, are associated with environmental exposures and could serve as a biomarker of current and/or past exposure regardless of whether they are mechanistically involved in the disease process [2].

The use of DNAm profiles as biomarkers of environmental exposures can provide opportunities to assess environmental influences on health outcomes that may otherwise not be possible when there are challenges with measures of exposure. For example, an epigenetic biomarker may serve as a surrogate for unmeasured prior exposure in cases where exposure information was not collected. DNAm patterns have been shown to reflect past exposures to prenatal smoking, prenatal alcohol, exposure to pesticides and toxins, maternal diet, and other health metrics [3]. DNAm signatures may also capture exposure timing and act as a personal dosimeter of exposure levels. Interview- and record-/report-based methods of exposure ascertainment can be subject to bias and are not always an accurate means of assessing the quantity, frequency, or timing of exposure. Methylation profiles may provide more specificity and greater accuracy of information on previous exposures. Epigenetic biomarkers may also capture interindividual differences in response to exposure, serving as a tool for assessing outcomes. Finally, as a biomarker of exposure, DNAm signatures may also serve as a metric to evaluate the effectiveness of exposure interventions. Although DNAm has been used previously in the study of substances and toxicants, these biomarkers can be extended to capture information about socioeconomic position (SEP). The purpose of this review is to (i) define SEP, (ii) provide evidence to support the use of DNAm as a biomarker of SEP, and (iii) present a framework for the use of DNAm biomarkers as a proxy and/or mediator in studies of health and development.

## SEP and Health Outcomes

One potential application of DNAm biomarkers is in capturing how social determinants of health become embodied [4]. Socioeconomic position (SEP), or one’s position in society that determines access to resources and services for well-being, may be expressed biologically. SEP is an extension of the more commonly known construct of socioeconomic status (SES). SEP is distinct from SES in that SES refers to one’s social ranking in society, while SEP encompasses both social ranking in society and resources that are available to that person [4, 5]. In that way, SEP is an aggregate factor that relates to socially derived economic factors that influence the position individuals hold in a multiple-stratified society [6]. We use SEP throughout this paper, as we are considering the totality of social ranking and tangible resources as potential pathways to DNAm.

A large body of literature suggests that SEP is strongly associated with mortality and morbidity in multiple health domains [7]. Lower SEP is associated with an increased risk of chronic health problems such as cardiovascular disease, diabetes, hypertension, respiratory infections, and cancer [8], while higher SEP is associated with better health outcomes.

SEP is hypothesized to act via multiple mechanisms in manifesting biological risk for disease. For instance, the material hypothesis suggests that access to resources such as health care, health education, housing, and food quality translates to better health outcomes [9]. Others theorize that low SEP operates through stress-related pathways via the hypothalamic–pituitary–adrenal axis to produce increased stress levels [10, 11]. In response to chronic stress, there is a complex physiological response whereby pro-inflammatory cytokines are released and serve as a negative feedback loop, extinguishing the hypothalamic–pituitary–adrenal axis and dampening the immune response [12–15]. Lower SEP is associated with systemic inflammation as measured by markers such as C-reactive protein and circulating interleukin-6, predisposing individuals to chronic inflammatory conditions such as autoimmune conditions, diabetes, cardiovascular disease, and cancer [16].

## Measures of SEP

SEP can be measured in different ways and at various levels, from individual to neighborhood. Some studies have used individual proxy factors, such as education, income, occupation, or subjective social ranking, while others have used composite scores [17]. There is no single best indicator of SEP [6], as each of these factors is related but represents unique measures capturing various aspects of SEP.

Education is one of the most widely used factors to measure SEP; higher levels of education are associated with better jobs, income, and material resources [18]. In childhood, parental education is often used as a proxy for SEP and is associated with the quality of a child’s environment and the health-related behaviors of the parents [19]. Income more precisely defines an individual’s ability to acquire material resources than education and is associated with better health in a non-linear relationship in most settings [20]. However, income is a dynamic variable that changes across the life course and thus may have variable effects on health at different time points [21]. Occupation is also used as a measure of SEP, given the relationship between job types and access to resources, education, and prestige. It has long been associated with health and mortality [22]. Because education, income, and occupation each capture correlated information about SEP but are not direct measures, many studies have used a composite SEP score. For example, the Hollingshead Four Factor Scale [23] is an index that measures a summative score of parental education level, parental occupation, marital status, and employment status. However, limitations to composite measures exist as changes in each SEP measure may affect outcomes differentially [24].

Neighborhood-level composite scores can also be summarized at a geographic level, such as the median income of a census tract or the percentage of those with high school education in a county. Neighborhood factors such as infrastructure deprivation, stress, and lack of social support can have significant impacts on morbidity and mortality [25].

Each of the individual- and neighborhood-level SEP indicators may have varied effects on health outcomes. While some have suggested that DNAm may be a harmonizable accessible metric to study the many ways in which SEP is embodied, a recent review by Cerruti *et al*. (2021) concluded that this may not be the case with little overlap in DNAm across SEP indicators [26]. However, given the methodological differences across prior studies, the potential for a biomarker capturing a common biological signature across SEP domains cannot be overlooked.

## DNAm as a Measure of SEP

Increasing evidence shows that SEP is associated with DNAm patterns in genes involved in stress and inflammation [27, 28] and those governing immune function [29–31]. In addition, there is human observational and model organism evidence that DNAm patterns reflect current, past, and cumulative exposures and could serve as a biomarker, even if it is not part of disease mechanisms. The best-studied evidence to date is for smoking exposure. Studies have identified a DNAm signature that can be detected in blood at birth, childhood, adolescence, and adulthood that reflects whether an individual had prenatal exposure to smoking [3], independent of personal history of smoking [32]. Other studies in adults have shown that DNAm patterns can distinguish between current and past smokers as well as those who never smoked, in addition to capturing cumulative measures of smoking, i.e. pack years [33]. Some of the changes observed in adult smokers appear to attenuate over time following smoking cessation but do not reach the same level as in non-smokers [33]. In a similar manner, DNAm has the potential to capture SEP exposure timing.

Cerruti *et al*. (2021) published a recent scoping review of 37 studies capturing global DNA, candidate gene studies, and epigenome-scale association studies (EWAS) to examine the association between SEP and DNAm in humans across the life course [26]. The main findings of the study suggested that SEP-related DNAm signatures varied by timing, duration, and type of SEP indicator. There was limited overlap, suggesting that different SEP domains, particularly education and income, have differential effects on DNAm. Moreover, the relationship between DNAm and SEP may differ depending on whether the exposure occurs in childhood or cumulatively over the lifespan. Since the publication of the review, Additional publications have examined life course SEP and DNA methylation, suggesting that socioeconomic disadvantage over the life course, not only in childhood or adulthood, is associated with epigenetic age acceleration, which was used as a biomarker of SEP-related health risk [34, 35]. Another study used a longitudinal design to show that changes in SEP, particularly worsening neighborhood quality and parental job loss in middle childhood, resulted in changes in DNAm at the age of 7, providing further evidence for socioeconomic mobility [36].

SEP-related factors are associated with specific DNAm patterns in EWAS (Table 1), candidate gene studies (Table 2), and global methylation studies (Table 3). Epigenetic signatures have been shown to capture SEP measures in the prenatal period, childhood, adulthood, and throughout the life course and have been observed in multiple tissue types, most commonly in blood/lymphocytes but also in the placenta, buccal samples, saliva, and adipose tissue. While the study by Cerruti *et al*. (2021) provided a comprehensive scoping review of articles showing evidence of the association between DNAm and SEP, we provide references that demonstrate aspects of SEP that have been examined in EWAS, candidate genes, and global methylation studies and may be of potential use for biomarker development [26]. However, more work must be performed to evaluate the robustness of these markers. Specifically, as can be seen in Tables 1–3, there are several time points when DNAm biomarkers of SEP indicators are not captured, and there is a paucity of articles looking at the consistency of these measures across SEP domains, tissue types, and time periods.

**Table 1:** EWAS showing DNAm changes in relation to social exposures

		Timing of DNAm measure			
SES/SEP-associated domain	Timing of exposure	Birth	Childhood	Adolescence	Adulthood
Composite score	Prenatal	‘Cord’: Laubach *et al*., 2019 [40]; ‘Placenta’: Santos *et al*., 2019 [39] ‘(no consistent gene loci across tissues)’	‘Blood’: Laubach *et al*., 2019 [40]		
	Childhood		‘Buccal’: Bush *et al*., 2018 [58]	‘Blood’: Beach *et al*., 2016 [60]	‘Blood’: Borghol *et al*., 2012 [29]
Parental education	Prenatal	‘Cord’: Alfano *et al*., 2018 [41]		‘Blood’: Alfano *et al*., 2018 [41]	
Parental occupational prestige	Childhood				‘Blood’: Lam *et al*., 2012 [61]
Household assets	Childhood				‘Blood’: McDade *et al*., 2017 [62]
Neighborhood disadvantage and financial stress	Childhood		‘Blood’: Dunn *et al*., 2019 [59]		
Educational attainment	Adulthood				‘Blood’: van Dongen *et al*., 2018; Karlsson Linnér *et al*., 2017 ‘(overlapping sample with multiple overlapping gene loci identified)’ [37, 38]

Papers were chosen using the following EMBASE search keywords, with selection of papers that included: (i) an SEP indicator, (ii) measured DNAm, and (iii) association results between them. Example keywords: “‘dna methylation’/exp OR ‘dna hypermethylation’:ab, ti, kw OR ‘dna hypomethylation’:ab, ti, kw OR ‘methylated deoxyribonucleic acid’:ab, ti, kw OR ‘methylated dna’:ab, ti, kw OR ‘dna methylation’:ab, ti, kw”; “‘social status’/exp OR ‘social condition*’:ab, ti, kw OR ‘social economic status’:ab, ti, kw OR ‘social importance’:ab, ti, kw OR ‘social rank’:ab, ti, kw OR ‘social standing’:ab, ti, kw OR ‘social state’:ab, ti, kw OR ‘socioeconomic status’:ab, ti, kw OR ‘socioeconomic status’:ab, ti, kw”; “‘biological marker’/exp OR ‘biological marker*’:ab, ti, kw OR ‘biomarker*’:ab, ti, kw.”

**Table 2:** Candidate gene studies showing DNAm changes in relation to social exposures

		Timing of DNAm measure (associated gene symbol)		
SES/SEP-associated domain	Timing of exposure	Birth	Childhood	Adulthood
Composite score	Prenatal	‘Cord’: King *et al*., 2015 (IGF2^a^, H19^a^, MEG3^a^) [63]; ‘Placenta’: Appleton *et al*., 2013 (HSD11B2) [64]	‘Buccal’: Piyasena *et al*., 2016 (IGF2^a^; FKBP5) [65]	
	Childhood			‘Adipose tissue’: Loucks *et al*., 2016 [69] (ASN, STAT3 and TMEM88 in females, and NRN1 in males)
	Adulthood			‘Blood’: Jones-Mason *et al*., 2016 (SLC6A4) [70], Simons *et al*., 2017 (OXTR) [71] ‘Saliva’: Swift-Scalan *et al*., 2014 (COMT) [72]
Maternal education	Birth			‘Blood’: Huang, 2016 (HSD11B2) [73]
	Childhood		‘Blood’: Obermann-Borst *et al*., 2012 (INSIGF) [66], Obermann-Borst *et al*., 2013 (LEP) [67]	‘Blood’: Needham, 2015 [27] (4 out of 7 stress-related genes (AVP, BDNF, FKBP5, and OXTR) and 3 out of 11 inflammation-related genes (CCL1, CD1D, and F8)
Neighborhood disadvantage	Prenatal	‘Cord’: King *et al*., 2015 (MEG3^a^)		
	Childhood		‘Buccal’: Wrigglesworth *et al*., 2019 (BDNF) [68]	
	Adulthood			*‘*Blood’: Smith *et al*., 2017 [28] (2 out of 7 stress-related genes evaluated (CRF, SLC6A4) and 2 out of 11 inflammation-related genes (F8, TLR1)
Adult educational attainment	Adulthood			‘Blood’: Needham *et al*., 2015 [27] (2 out of 7 stress-related genes (AVP and SLC6A4) and 5 out of 11 inflammation-related genes (CD1D, F8, KLRG1, NLRP12, and TLR3); de Rooij *et al*., 2012 [74] (GR 1-C)
Adult occupational position	Adulthood			‘Blood’: Stringhini *et al*., 2015 (NFATC1, PTGS2, CXCL2, ADM, IL1A, GPR132, MAP2K, TNFRSF11A, OLR1) [31]

Papers were chosen using the following EMBASE search keywords, with selection of papers that included (i) an SEP indicator, (ii) measured DNAm, and (iii) association results between them. Example keywords: “‘dna methylation’/exp OR ‘dna hypermethylation’:ab, ti, kw OR ‘dna hypomethylation’:ab, ti, kw OR ‘methylated deoxyribonucleic acid’:ab, ti, kw OR ‘methylated dna’:ab, ti, kw OR ‘dna methylation’:ab, ti, kw”; “‘social status’/exp OR ‘social condition*’:ab, ti, kw OR ‘social economic status’:ab, ti, kw OR ‘social importance’:ab, ti, kw OR ‘social rank’:ab, ti, kw OR ‘social standing’:ab, ti, kw OR ‘social state’:ab, ti, kw OR ‘socioeconomic status’:ab, ti, kw OR ‘socioeconomic status’:ab, ti, kw OR ‘socioeconomic position’:ab, ti, kw”; “‘biological marker’/exp OR ‘biological marker*’:ab, ti, kw OR ‘biomarker*’:ab, ti, kw.”

a Imprinted genes.

**Table 3:** Global methylation studies showing DNAm changes in relation to social exposures

		Timing of DNAm measure			
SES/SEP-associated domain	Timing of exposure	Birth	Childhood	Adolescence	Adulthood
Neighborhood-level deprivation	Prenatal	‘Cord’: Coker *et al*., 2018 (DNAm of LINE-1 and Alu repeat elements) [75]			
No association with SES/SEP measures	Prenatal		‘Blood’: Herbstman *et al*., 2013 (% Global DNAm) [76]		
Maternal education	Childhood		‘Blood’: Perng *et al*., 2012 (DNAm of LINE-1 elements) [77]		‘Blood’: Tehranifar *et al*., 2013 (Sat-2 elements) [78]
Family income	Childhood				‘Blood’: Tehranifar *et al*., 2013 (Sat-2 elements) [78]
Adult wealth	Adulthood				‘Blood’: Subramanyam *et al*., 2013 (DNAm of LINE-1 and Alu elements) [79]
Adult income					‘Blood’: Tehranifar *et al*., 2013 (DNAm of LINE-1 elements) [78]
Composite score education, occupation, neighborhood-level deprivation	Adulthood				‘Blood’: McGuinness *et al*., 2012 (global DNAm) [30]

Papers were chosen using the following EMBASE search keywords, with selection of papers that included: (i) an SEP indicator, (ii) measured DNAm, and (iii) association results between them. Example keywords: “‘dna methylation’/exp OR ‘dna hypermethylation’:ab, ti, kw OR ‘dna hypomethylation’:ab, ti, kw OR ‘methylated deoxyribonucleic acid’:ab, ti, kw OR ‘methylated dna’:ab, ti, kw OR ‘dna methylation’:ab, ti, kw”; “‘social status’/exp OR ‘social condition*’:ab, ti, kw OR ‘social economic status’:ab, ti, kw OR ‘social importance’:ab, ti, kw OR ‘social rank’:ab, ti, kw OR ‘social standing’:ab, ti, kw OR ‘social state’:ab, ti, kw OR ‘socioeconomic status’:ab, ti, kw OR ‘socioeconomic status’:ab, ti, kw”; “‘biological marker’/exp OR ‘biological marker*’:ab, ti, kw OR ‘biomarker*’:ab, ti, kw.”

Karlsson Linnér (2017) and van Dongen (2018) both assessed the same SEP domain, namely educational attainment, in adult blood with overlapping DNAm signatures (for a full summary of relevant CpG sites, see Supplementary Table S5 in Cerutti *et al*., 2021) [26, 37, 38]. However, these meta-analyses included overlapping samples, which may account for the consistency in findings. When examining non-overlapping samples, Cerutti *et al*. (2021) reported summary statistics compiled from nine EWAS showing 14 of 113 unique CpG sites associated with SEP domains across more than one study with 12 CpG sites in the education domain, one CpG site in the income domain, and one CpG site within the composite domain [26]. Studies investigating biomarkers owe careful consideration of the SEP construct, which requires standardization across studies as there can be nuanced biological and health impacts across different measures of SEP.

Another question is whether CpG sites for the same SEP domain are consistent across tissues within the same domain and tissue type. Santos *et al*. (2019) and Laubach *et al*. (2019) examined the relationship between a composite measure of SEP and methylation in placental and cord blood, respectively, but did not find any overlap in CpG sites that were significantly associated with DNAm at birth [39, 40]. The lack of consistency may reflect the differences in the individual factors used to create the composite score in these studies. Nonetheless, evidence is lacking at present that DNAm signatures are replicable across tissue types.

Finally, the consistency of findings can also be examined over time. Table 1 shows that Alfano *et al*. (2018) found two relevant CpG sites within the SULF1 gene that were significantly associated with maternal education at birth in cord blood as well as in adolescence [41]. However, there is a dearth of similar studies looking at longitudinal associations between DNAm and SEP indicators over the life course.

## Framework for the Use of Epigenetic Signatures of SEP in Health and Disease Research

DNAm signatures have several characteristics that make them ideal biomarkers to capture SEP as a measure of risk for health outcomes. First, DNAm is practical to collect in large samples across multiple collection sites and can be modified by changing environmental influences and across time [3]. Second, DNAm measures can also be measured in accessible tissues such as blood, urine, sperm, nasal, and stool samples. Third, methylation profiles of different SEP domains are unique, and therefore DNAm may have the potential to capture specific socioeconomic exposures. As evidence accumulates in the field of social epigenomics, there is increased interest in using DNAm signatures as a biomarker of disease. Here, we provide a framework for using epigenetic biomarkers of SEP in health and disease research.

**Figure 1:**
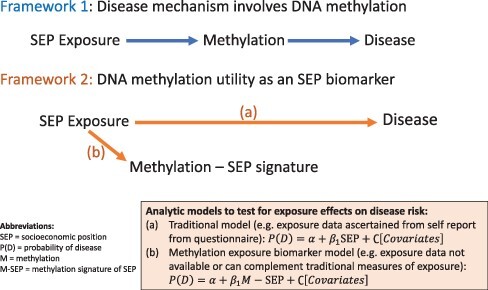
Conceptual framework and statistical models overview for epigenetic utility in exposure disease association analyses. M: methylation; P(D): probability of disease; S: methylation signature of SEP

Figure 1 provides a conceptual model to illustrate the difference between disease mechanism and exposure biomarker models. As shown, exposure biomarkers can be used to test the association between exposure and outcome to study questions on how SEP becomes biologically relevant.

As illustrated, the traditional model uses self-report questionnaires and other sources of information such as neighborhood census reports to delineate SEP. When such traditional exposure information is not available, or to complement traditional exposure measures, methylation-based models can be used as a surrogate.

We describe the steps for creating these methylation-based models briefly and specifically in the context of an SEP biomarker, although there is an extensive literature detailing the considerations for building prediction models in the broader context of epidemiological research [42, 43]. Notably, there are techniques and considerations for building prediction models in the field of statistical genetics [44, 45] that may apply to the field of epigenetics as methodology develops to fully leverage the high-dimensional data resulting from EWAS.

The first step to creating methylation-based models is to select CpG sites and their association statistics with SEP in a “training” population. This “training” population may consist of previous studies of SEP-related methylation changes, as delineated in Tables 1–3, or alternatively, an investigator may wish to extract novel associations using their own methylation platforms and populations of interest, or the union of both published and new data sources. It may be of interest to only select sites that have reached a certain significance threshold for their associations with SEP or to apply other restrictions that may maximize predictive ability such as ensuring there is no correlation between sites [45]. Following the extraction of CpG sites and the strength of their associations with SEP in the “training” population, an investigator can build a predictive model with this information to generate methylation-based SEP scores in a “test” population. Such models can capture SEP exposure using single relevant CpG sites, aggregate SEP methylation risk scores, or machine learning prediction models that assign individuals with cumulative biomarker scores proportional to their probability of belonging to higher or lower SEP categories based on their methylation signature [42, 46–48]. The “test” population may be a different population than the one from which the original association statistics with SEP were derived or a non-overlapping subset of the same population from which the original association statistics were derived. The former approach of testing the score in a separate population from the “training” population is considered preferable to the latter approach because it allows for increased generalizability across populations [49–51]. The final step is to assess the performance of the biomarker score using statistical techniques such as the area under the Receiver operating characteristic curve, the C-statistic, calibration measures, and/or the ability of the biomarker to differentiate between individuals with varying levels of SEP using generalized linear regression models [43, 45, 52].

If the investigator deems the predictive ability of the biomarker sufficient to act as a surrogate of SEP, the biomarker scores can be assessed as a single exposure for the SEP-related health outcome of interest along with covariates as delineated in the bottom portion of the conceptual Fig. 1. In disease mechanistic questions of whether the methylation signature mediates the association between the SEP and the health outcome, the scores from the methylation-based model can be designated as mediators of the traditional measure of SEP under a causal framework.

Once replicable and generalizable SEP DNAm signatures are developed across studies, these biomarkers can be adjusted for in analyses to help disentangle the differential effects of other environmental factors (e.g. toxicants and health behaviors) on health and development outcomes. Ultimately, these individual factors can also be added to the exposure biomarker model to examine the cumulative and interactive effects in conferring increased risk for poor health outcomes.

## Future Directions

Research is needed to elucidate epigenetic signatures that can capture SEP, at specific life stages, to provide a complementary measure of SEP for use in studies to examine the effects of SEP on health outcomes. Further work remains in developing harmonizable composite measures of SEP at both the individual and neighborhood levels that can be used across studies to help disentangle the complex relationship between SEP and other related environmental exposures. Research is also needed to elucidate the best tissue types for sampling DNAm changes related to socioeconomic exposures. In light of organismal research showing intergenerational and transgenerational transmission of epigenetic changes related to social disadvantage [53, 54], it would also be of interest to explore how SEP-related epigenetic mechanisms could potentially transmit across generations in humans.

A crucial step to generating valid and precise epigenetic biomarkers is a meta-analysis of epigenetic associations with SEP [55]. Except for meta-analyses of educational attainment in adults [37, 38], meta-analyses both within and between domains of SEP are currently lacking in the literature encompassing social epigenomics. An ideal meta-analysis would reveal the consistency of effect directions and magnitudes across sites [26]. It will be crucial to expand DNAm data banks of SEP measures to increase access to data derived from different tissue types, over varied age ranges, and with different experimental designs. This level of information is necessary to develop ideal biomarkers of SEP from DNAm measures.

To facilitate meta-analyses, EWAS for SEP must report comprehensive summary statistics beyond their statistically significant sites. Gathering comprehensive information across studies of epigenetic associations with SEP will improve the performance of the biomarkers by increasing confidence in the measurements of methylation and expanding the number of sites represented in the biomarker.

The heterogeneity of studies reporting relationships between SEP constructs and DNAm is cited as a barrier to meta-analyses [26]. However, if the measurement of DNAm is consistent across studies, such as increasingly common with the use of methylation arrays, a meta-analysis can be accomplished under the conditions of heterogeneous measures of SEP, tissue, populations, and timing. Techniques commonly used in statistical genetics to observe concordance of genetic effects across heterogeneous conditions, such as random-effect models and subset-based analyses, could be adapted for epigenetic meta-analyses of SEP [56, 57]. Furthermore, if loci are found to capture SEP in heterogeneous conditions, their inclusion in a biomarker would enhance the transferability of the biomarker across settings.

Epigenome-scale studies that offer an unbiased capture of biomarkers of SEP and can be measured in accessible tissues would provide a valuable tool for early detection and intervention toward mitigating SEP-based health disparities.
